# Fusion transcription factors for strong, constitutive expression of cellulases and xylanases in *Trichoderma reesei*

**DOI:** 10.1186/s13068-019-1575-8

**Published:** 2019-09-30

**Authors:** Christian Derntl, Robert L. Mach, Astrid R. Mach-Aigner

**Affiliations:** Institute of Chemical, Environmental and Bioscience Engineering, TU Wien, Gumpendorfer Strasse 1a, 1060 Vienna, Austria

**Keywords:** *Trichoderma reesei*, Xyr1, Ypr1, Transcription factor, Enzyme production, Glycerol

## Abstract

**Background:**

The filamentous ascomycete *T. reesei* is industrially used to produce cellulases and xylanases. Cost-effective production of cellulases is a bottleneck for biofuel production. Previously, different strain and process optimizations were deployed to enhance enzyme production rates. One approach is the overexpression of the main activator Xyr1 and a second is the construction of synthetic transcription factors. Notably, these genetic manipulations were introduced into strains bearing the wild-type *xyr1* gene and locus.

**Results:**

Here, we constructed a Xyr1-deficient strain expressing a non-functional truncated version of Xyr1. This strain was successfully used as platform strain for overexpression of Xyr1, which enhanced the cellulase and xylanase production rates under inducing conditions, with the exception of lactose—there the cellulase production was severely reduced. Further, we introduced fusion transcription factors consisting of the DNA-binding domain of Xyr1 and the transactivation domain of either Ypr1 or Ypr2 (regulators of the sorbicillinoid biosynthesis gene cluster). The fusion of Xyr1 and Ypr2 yielded a moderately transactivating transcription factor, whereas the fusion of Xyr1 and Ypr1 yielded a highly transactivating transcription factor that induced xylanases and cellulases nearly carbon source independently. Especially, high production levels of xylanases were achieved on glycerol.

**Conclusion:**

During this study, we constructed a Xyr1-deficient strain that can be fully reconstituted, which makes it an ideal platform strain for Xyr1-related studies. The mere overexpression of Xyr1 turned out not to be a successful strategy for overall enhancement of the enzyme production rates. We gained new insights into the regulatory properties of transcription factors by constructing respective fusion proteins. The Xyr1–Ypr1-fusion transcription factor could induce xylanase production rates on glycerol to outstanding extents, and hence could be deployed in the future to utilize crude glycerol, the main co-product of the biodiesel production process.

## Background

The mesophilic ascomycete *Trichoderma reesei* (teleomorph *Hypocrea jecorina* [1]) is widely used for the industrial-scale production of cellulases and xylanases [2, 3]. These enzymes are an important aspect of the natural lifestyle of *T. reesei* as a saprotroph [4–6] and find a broad range of industrial applications in the food and feed industry, the textile industry, the pulp and paper industry, and for the production of lignocellulosic bioethanol [3, 7, 8]. In nature, *T. reesei* thrives on dead plant material by breaking down the cellulosic and hemicellulosic parts of the plant cell walls. A cost-effective production of cellulases (and xylanases) is thought to be a bottleneck for biofuel production. Consequently, strain design and process optimizations have been deployed with the aim to produce high amounts of cellulases (and xylanases) using cheap substrates.

Years of strain development led on the one hand to high yield production strains [2, 9], and on the other hand to a fundamental understanding of the plant cell wall-degrading enzymes (PCWDEs) and the responsible regulation machinery [5, 10, 11]. The main PCWDEs are the two cellobiohydrolases CBHI and CBHII (EC 3.2.1.91), the endoglucanase EGLI (EC 3.2.1.4), the β-glucosidase BGLI (EC 3.2.1.21), the two endo-β-1,4-xylanases XYNI and XYNII (EC 3.2.1.8), and the β-xylosidase BXLI (EC 3.2.1.37).

The two main regulators of cellulase and hemicellulase expression are the C2H2 zinc finger protein Cre1, the mediator of carbon catabolite repression (CCR) [12, 13], and the Gal4-like transcription factor (TF) Xyr1, which is essential for expression of nearly all PCWDEs [14] and the aldose reductase Xyl1 (EC 1.1.1.307) [15]. Over the years, several additional regulatory proteins and signal transduction pathways were described to be involved in the regulation of PCWDEs expression, e.g., the TFs Ace1 [16], Ace2 [17], Ace3 [18], Xpp1 [19] and Rce1 [20], the mating-type locus protein Mat1-2-1 [21], the photoreceptor Env1 [22], the protein methyltransferase Lae1 [23], the velvet complex protein Vel1 [24], and the MAP kinases Tmk2 [25] and Tmk3 [26].

However, the central role of Xyr1 remained unchallenged over the years. Expression of Xyr1 itself is induced by cellulase inducing conditions (sophorose, lactose) by yet unidentified mechanisms and repressed by glucose and high concentrations of d-xylose as part of the CCR [27–29]. High expression levels of Xyr1 were observed simultaneously with high expression levels of cellulases and a direct causal linkage between them was suggested [27–30]. Consequently, overexpression of Xyr1 was performed to enhance cellulase production, using the wild-type Xyr1 [31] or a mutated version [32]. In recent reports, the utilization of fusion TFs in *T. reesei* was described. A fusion of Cre1 and Xyr1 resulted in enhanced cellulase production on glucose in CCR-released Rut-C30 [33]. In contrast, a fusion of the VP16 activation domain to the complete Xyr1 abolished cellulase production on lactose and Avicel [34]. The authors speculate that the fusion TF might interact with the wild-type Xyr1 and thus form non-functional heterodimers. Notably, all these experiments were performed in strains that still contained and expressed the wild-type Xyr1. We assume that this circumstance is based on the fact that the cellulolytic and xylanolytic activities cannot be fully reconstituted in *xyr1* deletion strains, neither by ectopic integration of *xyr1* nor by reestablishment of the original locus (unpublished results by ARMA and RLM).

However, in a previous study, we could demonstrate that a single point mutation in the Fungal Transcription Factor Middle Homology Region (FTFMHR) of Xyr1 leads to a glucose blind phenotype in industrial strains of *T. reesei* with completely deregulated *xyn2* expression [29]. A partial deletion and mutation analysis of XlnR, the homolog of Xyr1 in *Aspergillus niger,* led to similar results; the authors suggested that auto-regulation plays an important role in the function of XlnR [35]. This might also be true for Xyr1 considering the similar biological function and the high sequence and structural similarity of the two Gal4-like TFs.

Unrelated, we have studied the gene cluster responsible for the formation of a typical yellow pigment in *T. reesei* [36, 37]. Sorbicillinoids are a group of yellow secondary metabolites, more precisely polyketides that are produced by several filamentous fungi of different genera including *Trichoderma* [38] and *Penicillium* [39]. They are named after the hexaketide sorbicillin, which was the first described sorbicillinoid, originally isolated from *P. chrysogenum* [39]. Please refer to two reviews about sorbicillinoids by Harned et al. [40] and Meng et al. [41]. Meanwhile, new findings have been gained regarding the biosynthetic pathway in *T. reesei* [37] and *P. chrysogenum* [42, 43]. However, the sorbicillinoid gene cluster in *T. reesei* contains two Gal4-like TFs, Ypr1 and Ypr2 (Yellow pigment regulator 1 and 2). Ypr1 is the main activator of the cluster and Ypr2 mediates a negative feed-back loop regulation [36]. It remained unclear whether Ypr2 is acting directly as a repressor or activates transcription of an additional repressor [36]. However, in *P. chrysogenum,* a similar feedback mechanism was proposed for the Ypr2 homolog [42].

In this study, we constructed a strain bearing a non-sense point mutation in Xyr1 that can be used for reconstitution of Xyr1 expression, and thus serves as an ideal platform for Xyr1-related investigations. We sequenced the coding regions of *ypr1* and *ypr2* and performed an in silico analyses of the two Gal4-like TFs Ypr1 and Ypr2 and compared them to each other and to the Gal4-like TF Xyr1. Based on the sequence comparisons, we constructed fusion TFs consisting of the DNA-binding domain of Xyr1 and the transactivation domain of Ypr1 or Ypr2 and inserted them into the mentioned Xyr1-deficient *T. reesei* strain. In the resulting strains, cellulase and xylanase activities and the transcript levels of the main PCWDE-encoding genes were measured after cultivation on different carbon sources.

## Results

### Construction of a Xyr1-deficient strain that can be reconstituted

**Fig. 1:**
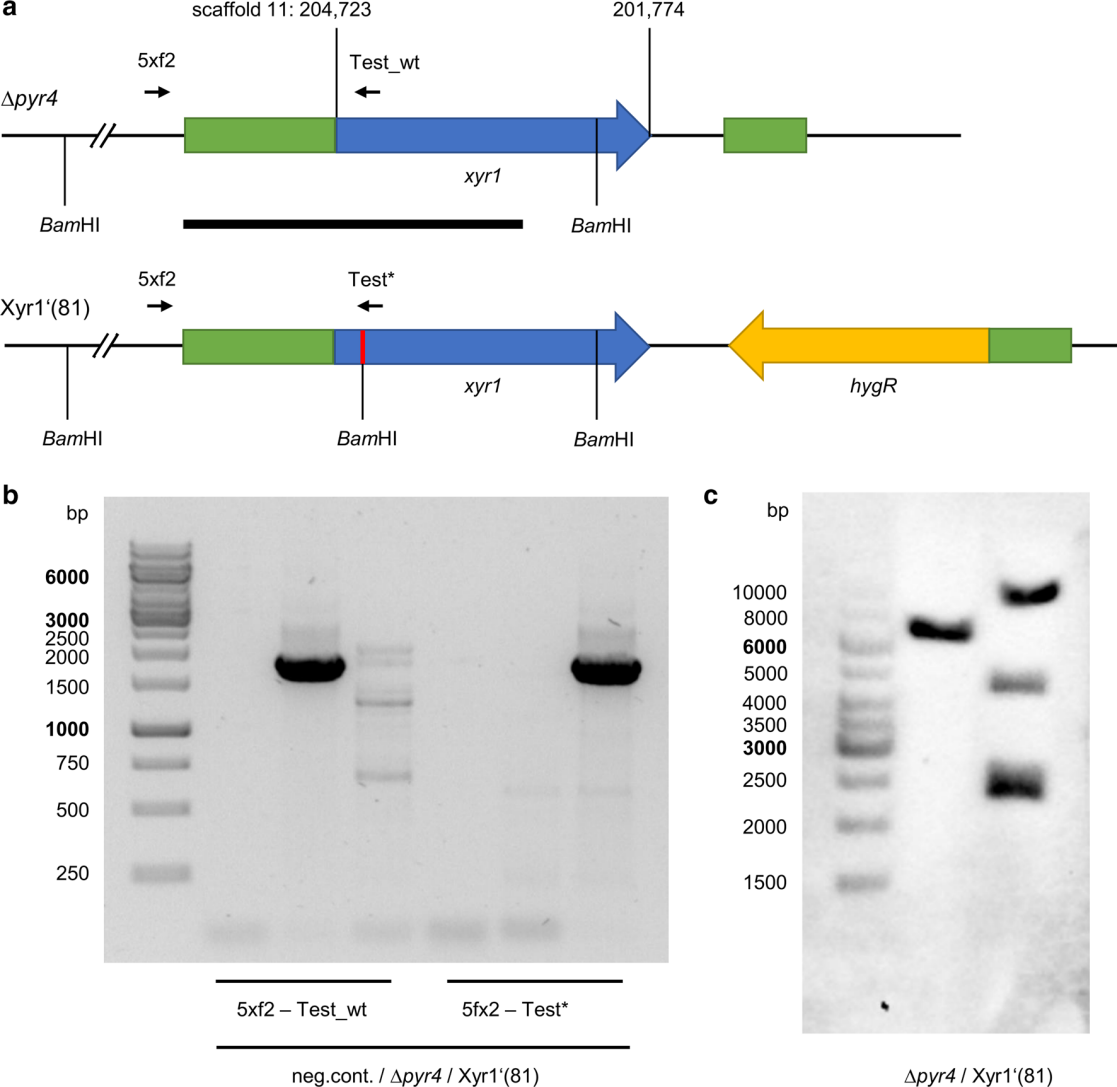
Construction of the Xyr1-deficient *T. reesei* strain Xyr1′(81). **a** The uridine auxotrophic strain Δ*pyr4* was transformed with the plasmid pCD-Xyr1′(81)-HR, resulting in the insertion of a non-sense mutation (red line) and an adjacent *Bam*HI restriction site in the *xyr1* gene (blue arrow). The indicated flanking regions (green boxes) and the hygromycin resistance cassette (yellow arrow) were used for the homologous replacement strategy. Genomic coordinates are given on top. Position and orientation of the primers used for genomic testing are indicated by the short, black arrows. 5xf2, 5Xyr1_fwd2; Test_wt, Xyr1wt_Test_250rev; Test*, Xyr1*_Test_250rev. The thick, black line indicates the hybridization region for the probe used in the Southern blot assay. **b** Agarose gel electrophoresis of PCRs using the indicated primers and genomic DNA of indicated strains were performed to verify the complete replacement of the endogenous *xyr1* gene. **c** A Southern blot analysis using *Bam*HI-digested chromosomal DNA of the indicated strains and the indicated probe returned the expected signals at 6370 bp for Δ*pyr4* and 4170 bp and 2200 bp for Xyr′(81), along with an additional band above 10,000 bp indicating an ectopic insertion of the replacement cassette in Xyr1′(81) somewhere else in the genome

**Fig. 2:**
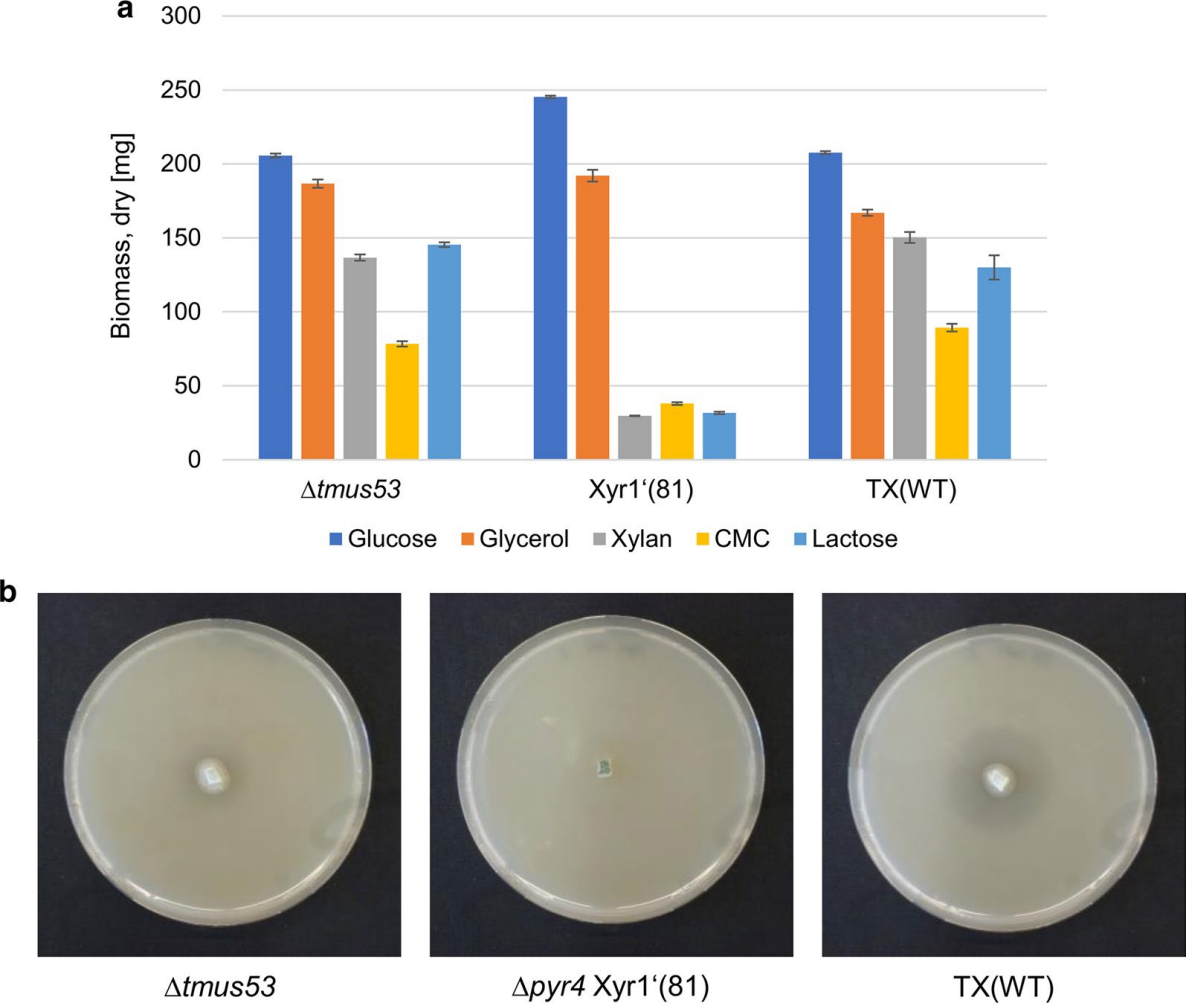
Influence of Xyr1 on growth behavior and xylanolytic properties in *T. reesei*. **a** The wild-type-like Δ*tmus53*, the Xyr1-deficient Xyr1′(81), and the Xyr1-overexpression TX(WT) *T. reesei* strains were cultivated on the indicated carbon sources for 72 h and the dry weight of the accumulated biomass measured. The cultivation was performed in triplicates. Values are means; error bars represent the standard deviations. **b** The wild-type-like Δ*tmus53*, the Xyr1-deficient Xyr1′(81), and the Xyr1-overexpression TX(WT) *T. reesei* strains were cultivated on xylan plates and pictures were taken after 72 h

Earlier, we observed that the deletion of *xyr1* leads to a complete abolishment of the expression of most PCWDEs in *T. reesei* and to a strongly reduced growth on d-xylose [14]. The ectopic integration of a *xyr1* expression cassette into this strain did only restore the ability to grow on d-xylose but not the expression of the PCWDEs (unpublished observations). In this study, we followed an alternative approach; we introduced a non-sense point mutation at the N-terminus of Xyr1, with the aim to obtain a Xyr1-deficient strain that only has a minor genetic difference. To this end, we transformed the plasmid pCD-Xyr1′(81)-HR into *T. reesei* Δ*pyr4* to introduce a non-sense point mutation at position 81 (5′-AAG-3′ encoding for lysine was replaced with the stop codon 5′-TAG-3′) using a homologous replacement strategy (Fig. 1a) yielding the strain *T. reesei* Xyr1′(81). We confirmed the correct integration of the replacement cassette at the *xyr1* locus and the absence of any wild-type *xyr1* by PCR (Fig. 1b). A Southern blot analysis confirmed the complete replacement at the *xyr1* locus (Fig. 1c). We observed an additional signal in *T. reesei* Xyr1′(81), which suggests that the replacement cassette was inserted ectopically at a second locus (Fig. 1c). Despite, we decided to proceed with this strain because it had the desired Xyr1-deficient phenotype, i.e., it can hardly grow on xylan, CMC, and lactose (Fig. 2a) and the xylanolytic activity is abolished on xylan plates (Fig. 2b). Notably, *T. reesei* Xyr1′(81) still bears the *pyr4* deletion; this is the basis for the subsequent targeted gene insertions as described in a previous publication [44].

**Fig. 3:**
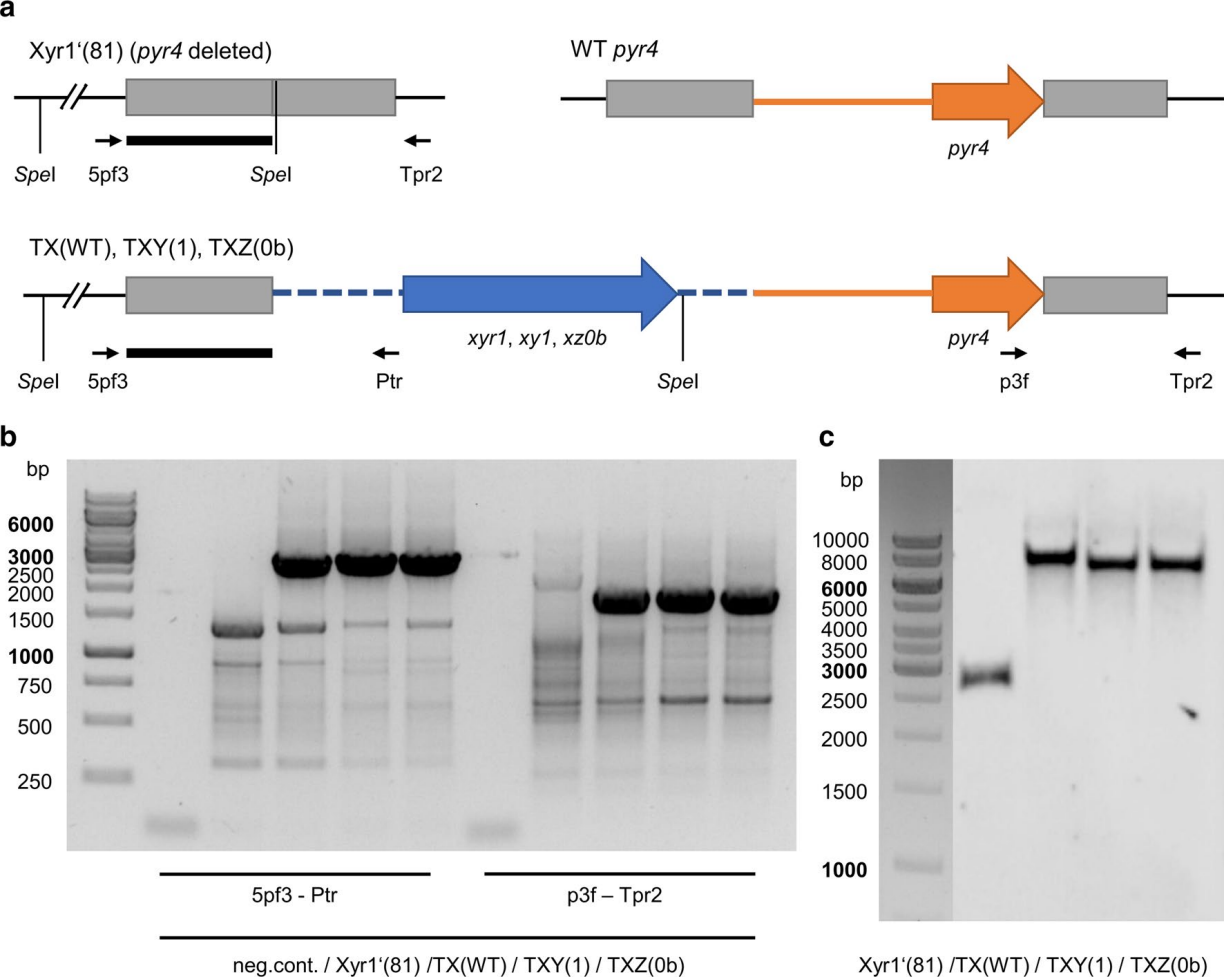
Genomic insertion of Xyr1, XY1, and XZ0b expression cassettes. **a** The uridine auxotrophic strain Xyr1′(81) was transformed with the plasmids pRP4-TX(WT), pRP4-TXY(1), or pRP4-TXZ(0b) resulting in the targeted integration of the respective expression cassettes (blue arrow and blue, dashed lines) into the *pyr4* locus using the *pyr4* gene (orange arrow) and its promoter (orange line) as auxotrophic marker. The gray boxes represent the flanking regions used for the homologous recombination strategy. The wild-type *pyr4* locus is depicted for comparison only. Position and orientation of the primers used for genomic testing are indicated by the short, black arrows. 5pf3, 5pyr4_fwd3; Ptr, Ptef_rev-BspTI; p3f, pyr4_3fwd; Tpr2, Tpyr4_rev2. The thick, black line indicates the hybridization region for the probe used in the Southern blot assay. Recognition sites for the restriction endonuclease *Spe*I are depicted. **b** Agarose gel electrophoresis of PCRs using the indicated primers and genomic DNA of indicated strains were performed to verify the integration of the TF expression and *pyr4* re-establishment cassettes into the *pyr4* locus. **c** A Southern blot analysis using *Spe*I-digested chromosomal DNA of the indicated strains and the indicated probe returned the expected signals at 2501 bp for Xyr1′(81) and 6670 bp, 6355 bp, and 6274 bp for TX(WT), TXY(1), and TXZ(0b), respectively

Next, we wanted to test whether the transactivating activity of Xyr1 can be reconstituted in this strain. We transformed the plasmid pRP4-TX(WT) into *T. reesei* Xyr1′(81) with the aim to insert the Xyr1 expression cassette into the *pyr4* locus (Fig. 3a) resulting in the strain *T. reesei* TX(WT). We confirmed the correct and exclusive integration of the expression cassette at the *pyr4* locus by PCR and Southern blot analysis (Fig. 3b, c). *T. reesei* TX(WT) regained the ability to grow on xylan, CMC, and lactose (Fig. 2a) and the xylanolytic activity was reconstituted on xylan plates (Fig. 2b). Hence, we conclude that *T. reesei* Xyr1′(81) is a suitable platform strain for Xyr1 expression studies.

### Overexpression of Xyr1 leads to enhanced xylanolytic activity

**Fig. 4:**
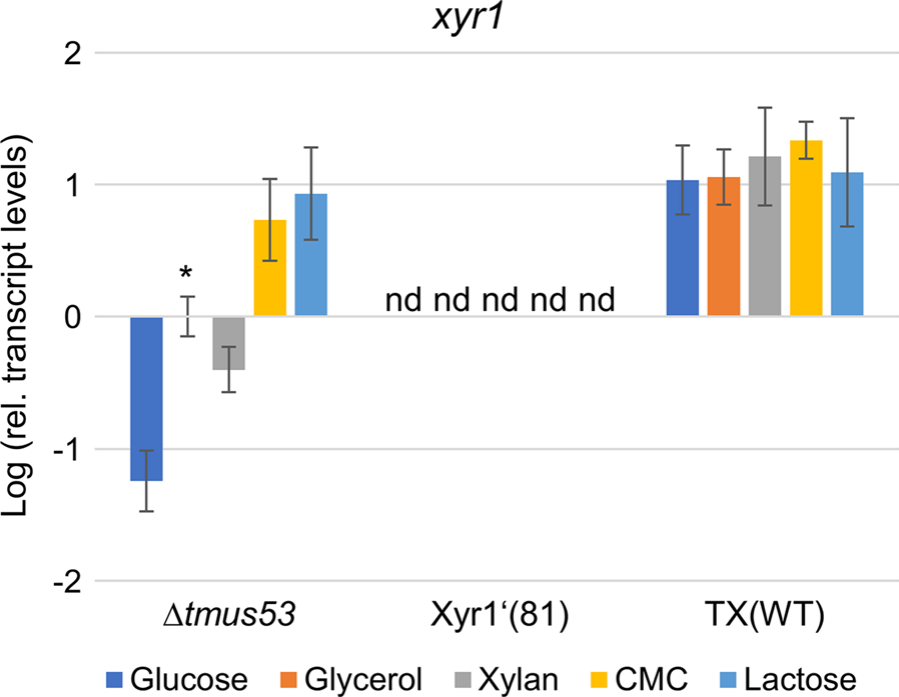
Transcript levels of *xyr1* in the overexpression strain TX(WT). The wild-type-like strain Δ*tmus53*, the Xyr1-deficient strain Xyr1′(81), and the Xyr1-overexpression strain TX(WT) were cultivated on the indicated carbon sources and samples were taken after 24 h (glucose, glycerol, xylan, lactose) or 48 h (CMC). Relative transcript levels of *xyr1* were measured by RT-qPCR analysis, normalized to the reference sample (Δ*tmus53*, glycerol, indicated by asterisk) using the reference genes *sar1* and *act1*. Mean values are given; error bars indicate standard deviation from three independently grown cultures

**Fig. 5:**
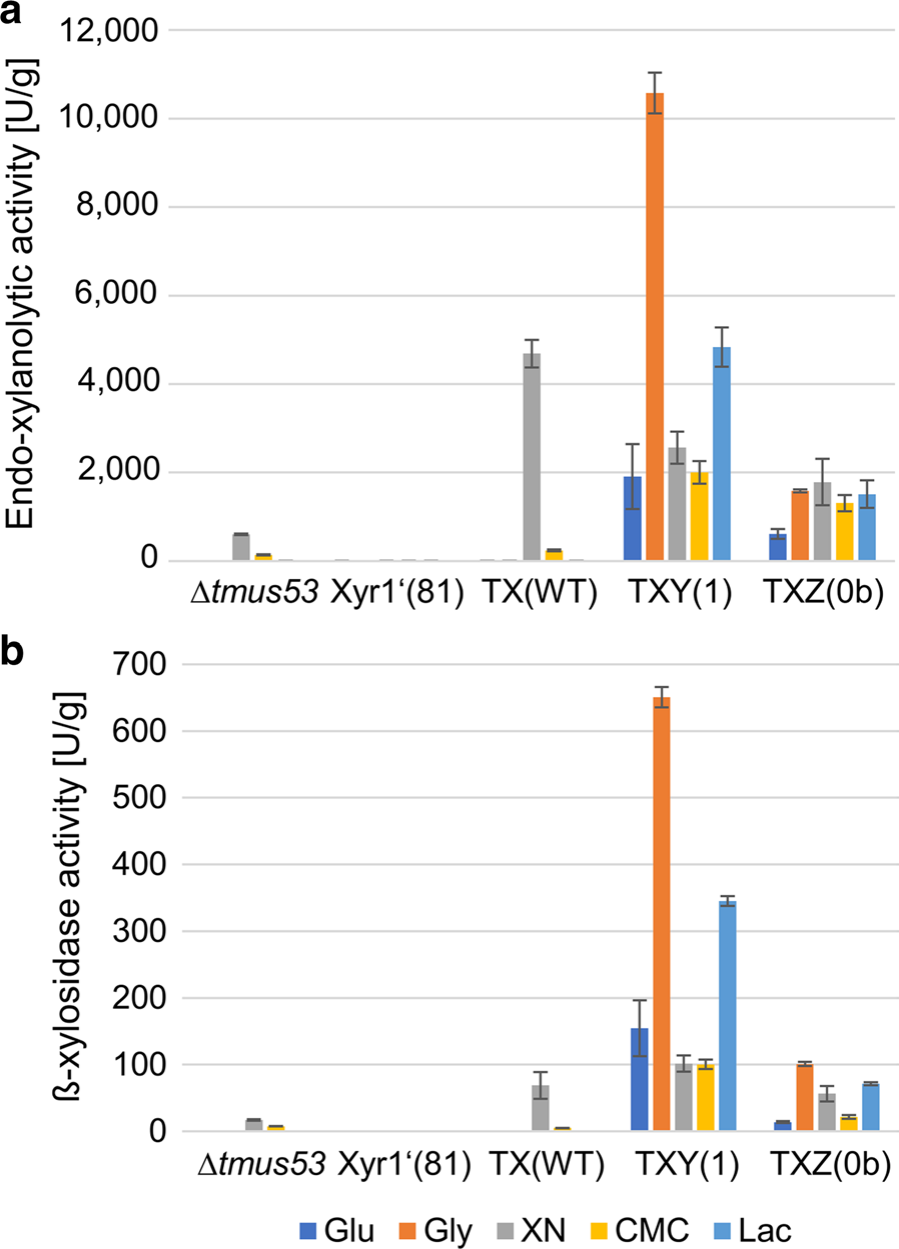
Influence of Xyr1 overexpression on xylanolytic activity. The wild-type-like strain Δ*tmus53*, the Xyr1-deficient strain Xyr1′(81), the Xyr1-overexpression strain TX(WT), and the fusion TF bearing strains TXY(1) and TXZ(0b) were cultivated on the indicated carbon sources and samples were taken after 72 h. The endo-xylanolytic activity (**a**) and the β-xylosidase activity (**b**) were measured in the supernatants and normalized to the acquired biomass. The values provided in the figures are means from three biological experiments. Error bars indicate standard deviations

*Trichoderma reesei* TX(WT) caused a larger clearing halo on the xylan plates than the wild-type-like strain *T. reesei* Δ*tmus53* (Fig. 2b), pointing towards higher xylanases expression rates. Notably, in *T. reesei* TX(WT), expression of Xyr1 is driven by the strong constitutive *tef1* promoter. This results in higher *xyr1* transcript levels compared to the wild-type-like strain *T. reesei* Δ*tmus53* (Fig. 4). The primers used in the RT-qPCR assay were designed to amplify only the wild-type *xyr1* transcript. To study the influence of the high *xyr1* transcript levels on the expression of xylanases in more detail, we cultivated the wild-type-like strain Δ*tmus53*, the Xyr1-deficient strain Xyr1′(81), and the Xyr1-overexpression strain TX(WT) on different carbon sources for 72 h and measured the endo-xylanolytic activities in the resulting supernatants using Azo-xylan and the β-xylosidase activity using *p*-nitrophenyl β-d-xylopyranoside. We used the repressing carbon source glucose, glycerol which is considered to be neutral (not repressing, not inducing), xylan which induces xylanases expression and the two cellulase expression-inducing carbon sources, CMC and lactose. As expected, no xylanolytic activities could be detected in Xyr1′(81) on all carbon sources (Fig. 5a, b). In the wild-type-like Δ*tmus53* and the Xyr1-overexpression strain TX(WT), xylanolytic activities could only be measured on xylan and to minor extent also on CMC (Fig. 5a, b). The overexpression of Xyr1 resulted in higher endo-xylanolytic activity (approx. 7.5-fold on xylan and 1.5-fold on CMC), and higher ß-xylosidase activity (approx. fourfold on xylan) compared to the wild-type-like Δ*tmus53.*

### Influence of the Xyr1 overexpression on cellulolytic activity

**Fig. 6:**
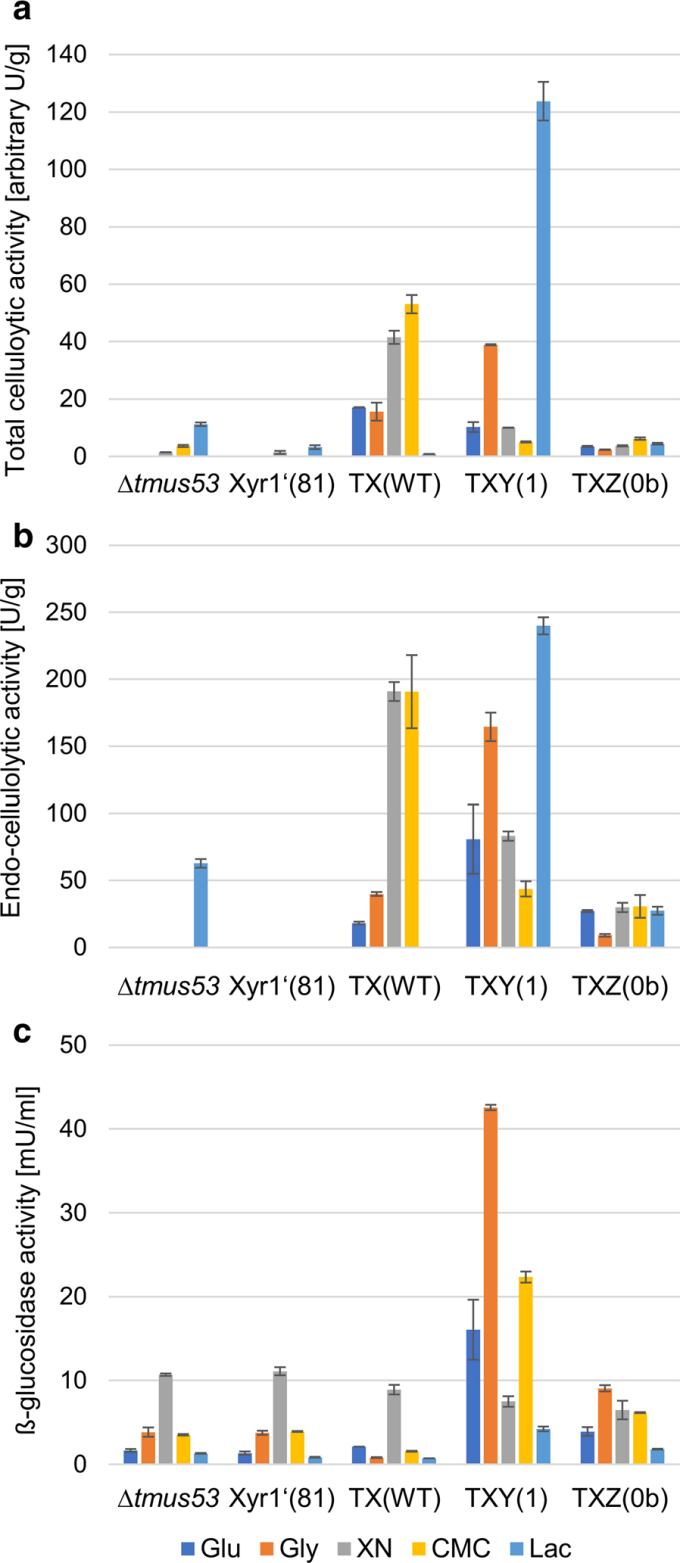
Influence of Xyr1 overexpression on cellulolytic activity. The wild-type-like strain Δ*tmus53*, the Xyr1-deficient strain Xyr1′(81), the Xyr1-overexpression strain TX(WT), and the fusion TF bearing strains TXY(1) and TXZ(0b) were cultivated on the indicated carbon sources and samples were taken after 72 h. The total cellulolytic activity (**a**), the endo-cellulolytic activity (**b**) and the β-glucosidase activity (**c**) were measured in the supernatants. The total cellulolytic and the endo-cellulolytic activities were normalized to the acquired biomass. The values provided in the figures are means from three biological experiments. Error bars indicate standard deviations

Next, we were interested in how the Xyr1 overexpression influences the cellulolytic activity in the Xyr1′(81) background. To this end, we measured the total cellulolytic activity, the endo-cellulolytic activity, and the ß-glucosidase activity in the above-described cultivation supernatants using resorufin-cellobioside, Azo-CMC, and *p*-nitrophenyl β-d-glucopyranoside, respectively. Notably, the obtained total and endo-cellulolytic activities on CMC have to be evaluated critically because the remaining CMC in the supernatant can compete with the test substrates. *T. reesei* Xyr1′(81) produced only very low levels of total cellulolytic activity on xylan and lactose (Fig. 6a), and no endo-cellulolytic activity at all (Fig. 6b). In the wild-type-like Δ*tmus53*, we could detect total cellulolytic activity on lactose and to some extent also on CMC (Fig. 6a). In contrast, endo-cellulolytic activity could only be measured on lactose, but not on CMC (Fig. 6b) although growth was observed there (Fig. 2a). In the Xyr1-overexpression strain TX(WT), we measured high total and endo-cellulolytic activity on xylan and CMC (Fig. 6a, b), and even activity on the neutral carbon source glycerol and the repressing carbon source glucose (Fig. 6a, b). Surprisingly, we could hardly detect any total cellulolytic activity and no endo-cellulolytic activity on lactose, despite normal growth (Fig. 2a) and high *xyr1* transcript levels (Fig. 4). We repeated this experiment with two independently generated strains and confirmed the unexpected results.

However, we could detect similar levels of β-glucosidase activity in the wild-type-like strain Δ*tmus53*, the Xyr1-deficient strain Xyr1′(81), and the Xyr1-overexpression strain TX(WT) on all tested carbon sources (Fig. 6c).

### Influence of Xyr1 overexpression on transcript levels of PCWDE-encoding genes

To gain a detailed insight on how the overexpression of Xyr1 influences the expression of the individual PCWDEs, we cultivated the wild-type-like strain Δ*tmus53*, the Xyr1-deficient strain Xyr1′(81), and the Xyr1-overexpression strain TX(WT) on glucose, glycerol, xylan, CMC, and lactose and took samples in the early stages of cultivation (48 h for CMC and 24 h for the others), when the induction in young mycelium is not yet overshadowed by the stagnant gene expression in old mycelium. Then, we isolated the total RNA from the samples and reverse transcribed the mRNA to perform qPCR assays. We determined the relative transcript levels for the PCWDE-encoding genes *cbh1*, *cbh2*, *egl1*, *bgl1*, *xyn1*, *xyn2*, and *bxl1* and the aldose reductase *xyl1*.

**Fig. 7:**
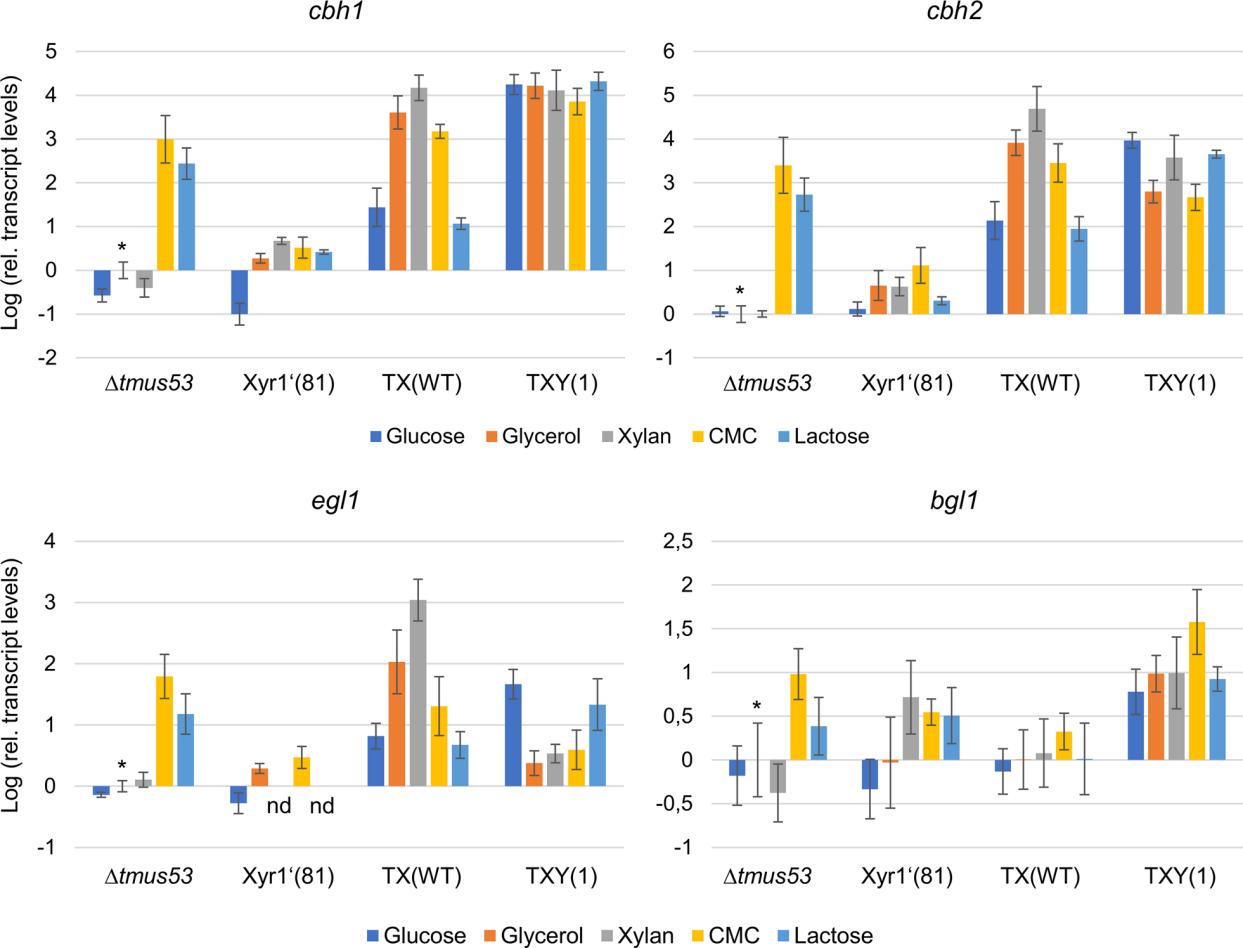
Transcript levels of the major cellulase-encoding genes. The wild-type-like strain Δ*tmus53*, the Xyr1-deficient strain Xyr1′(81), the Xyr1-overexpression strain TX(WT), and the fusion TF bearing strain TXY(1) were cultivated on the indicated carbon sources and samples were taken after 24 h (glucose, glycerol, xylan, lactose) or 48 h (CMC). Relative transcript levels of the indicated genes were measured by RT-qPCR analysis, normalized to the reference sample (Δ*tmus53*, glycerol, indicated by asterisk) using the reference genes *sar1* and *act1*. Mean values are given; error bars indicate standard deviation from three independently grown cultures

**Fig. 8:**
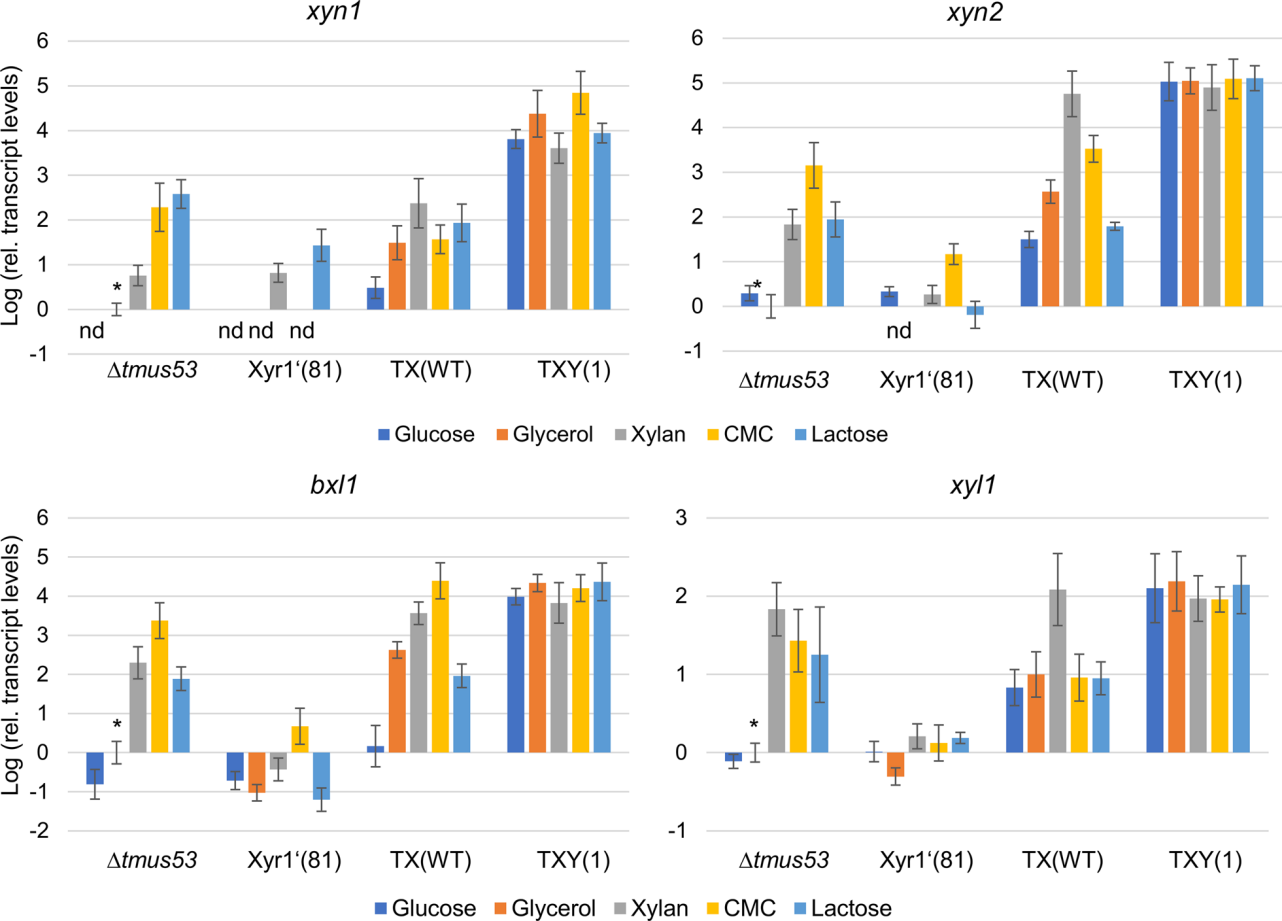
Transcript levels of the major xylanase-encoding genes and *xyl1*. The wild-type-like strain Δ*tmus53*, the Xyr1-deficient strain Xyr1′(81), the Xyr- overexpression strain TX(WT), and the fusion TF bearing strain TXY(1) were cultivated on the indicated carbon sources and samples were taken after 24 h (glucose, glycerol, xylan, lactose) or 48 h (CMC). Relative transcript levels of the indicated genes were measured by RT-qPCR analysis, normalized to the reference sample (Δ*tmus53*, glycerol, indicated by asterisk) using the reference genes *sar1* and *act1*. Mean values are given; error bars indicate standard deviation from three independently grown cultures

In the Xyr1-deficient strain, Xyr1′(81), basically none of the assayed genes were transcribed at elevated levels or could be detected at all (Figs. 7, 8). In the wild-type-like strain Δ*tmus53*, elevated transcript levels were measured for the cellulase-encoding genes, *cbh1*, *cbh2*, and *egl1* on CMC and on lactose, as expected (Fig. 7). On the other carbon sources (glucose, glycerol, and xylan) *cbh1*, *cbh2* and *egl1* were not or hardly transcribed (Fig. 7). In the Xyr1-overexpression strain TX(WT), the transcript levels of *cbh1*, *cbh2*, and *egl1* on CMC were similar to the levels in the wild-type-like strain Δ*tmus53* (Fig. 7). Notably, we could also detect high transcript levels of *cbh1*, *cbh2* and *egl1* on xylan and on glycerol, which was not observed in Δ*tmus53* (Fig. 7). On lactose, *cbh1*, *cbh2* end *egl1* were only transcribed at a low level in TX(WT) (Fig. 7), matching the unexpectedly low cellulolytic activity on lactose (Fig. 6a, b). They were in the same range as on glucose (Fig. 7). Notably, the *cbh1*, *cbh2,* and *egl1* levels on glucose were higher compared to Δ*tmus53* (Fig. 7). The *bgl1* transcript levels were at a similar, basal level in all three strains on all carbon sources (Fig. 7), matching the observed enzymatic activity (Fig. 6c).

The xylanase-encoding genes, *xyn1*, *xyn2,* and *bxl1* were strongly expressed in Δ*tmus53* on CMC (Fig. 8). Transcript levels of *xyn2* and *bxl1* were also elevated on xylan and lactose (Fig. 8). Unexpectedly, *xyn1* transcript levels were highly elevated on lactose but not on xylan (Fig. 8). No or only very low levels were detected on glucose and glycerol (Fig. 8). In TX(WT), *xyn1*, *xyn2*, and *bxl1* transcript levels were elevated on xylan, CMC, and on lactose (Fig. 8). Notably, elevated transcript levels were also detected on glycerol, in contrast to Δ*tmus53* (Fig. 8). On glucose, only low *xyn1* and *bxl1* and slightly elevated *xyn2* transcript levels were measured in TX(WT) (Fig. 8).

The transcription of the aldose reductase *xyl1*, which plays an essential role in the catabolism of d-xylose and lactose, was highly elevated on xylan, CMC, and lactose in both, Δ*tmus53* and TX(WT), being highest in TX(WT) on xylan (Fig. 8). In TX(WT), elevated levels were additionally measured on glucose and glycerol, in contrast to Δ*tmus53* (Fig. 8).

In summary, the non-sense mutation at position 81 in Xyr1 resulted in a Xyr1-deficient phenotype, i.e., the inability to grow on xylan, CMC, and lactose, and the abolishment of formation of cellulolytic and xylanolytic activity. In this genetic background, integration of a *xyr1*-overexpression cassette (at the *pyr4* locus) led to reconstitution of the lost abilities. However, the overexpression of Xyr1 did not cause generally enhanced cellulolytic and xylanolytic activity. Xylanolytic activity is only enhanced on xylan, and cellulolytic activities on glucose, glycerol, xylan, and CMC, but completely abolished on lactose. In other words, the carbon sources still influence the expression of the PCWDEs in a strain overexpressing Xyr1, suggesting that further mechanisms (e.g., autoregulation) and/or other TFs play important roles in the regulation of PCWDEs expression.

### In silico comparison of the Gal4-like transcription factors Xyr1, Ypr1 and Ypr2

We reasoned that replacing the FTFMHR of Xyr1 with a FTFMHR from another Gal4-like TF might overcome the assumed autoregulatory mechanism and any Xyr1-specific protein interactions (e.g. the proposed interaction with the mating-type locus protein Mat1-2-1 [21]) and or modifications. Ypr1, the main regulator of sorbicillinoid biosynthesis in *T. reesei,* is a Gal4-like TF consisting of only 674 amino acids (Xyr1 has 940 aa). This and its strong and very direct regulatory properties [36] make Ypr1 an ideal candidate for the FTFMHR replacement. The second Gal4-like TF from the sorbicillin gene cluster, Ypr2, consists of only 684 aa, but its regulatory properties remained undetermined [36]. To enable a knowledge-based fusion of protein domains, we performed a comparative in silico analysis of the TFs Xyr1, Ypr1, and Ypr2.

First, we determined the coding regions of *ypr1* and *ypr2* to obtain the corresponding primary structures of Ypr1 and Ypr2. To this end, we reverse transcribed the mRNA isolated from a sorbicillin producing culture sample of *T. reesei* (on glucose) and amplified the cDNA for *ypr1* and *ypr2* by PCR using primers based on the gene prediction models at the JGI Genome Portal (https://genome.jgi.doe.gov/Trire2/Trire2.home.html) [4]. We cloned the PCR products into the plasmid pJET1.2 and had 6 candidates each sequenced. The obtained sequences for *ypr1* and *ypr2* cDNA were deposited at the NCBI GenBank (accession numbers MN102104 and MN102105).

**Fig. 9:**
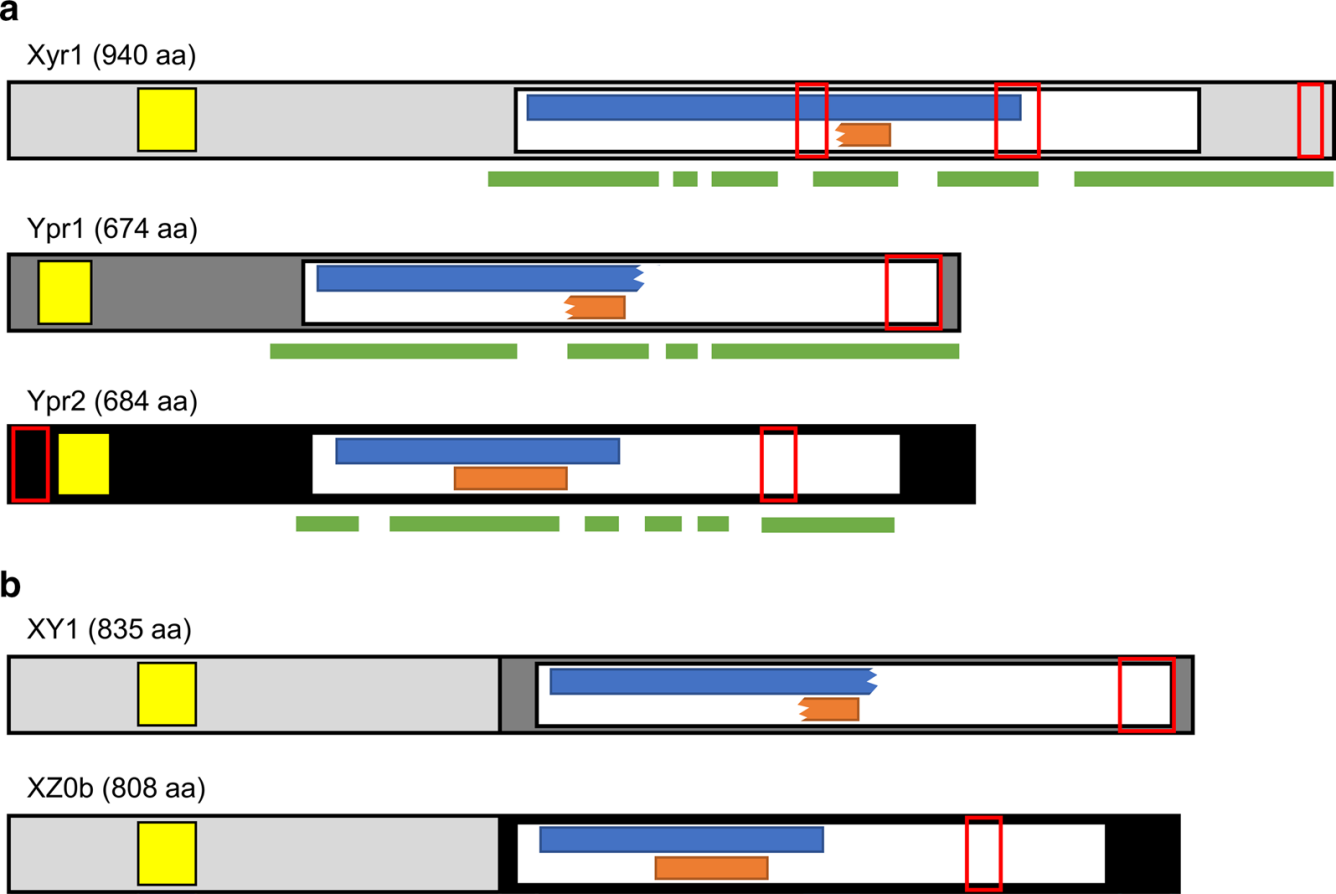
Schematic presentation of native and fusion TFs. **a** The primary structure of the three Gal4-like TFs, Xyr1, Ypr1, and Ypr2 was subjected to an in silico analysis. Yellow boxes, Zn(II)2Cys6 binuclear cluster DNA-binding domain (smart00066); white boxes, FTFMHR (cd12148); blue boxes, fungal-specific transcription factor domain pfam04082; orange boxes, fungal-specific transcription factor domain smart00906; red, empty boxes, predicted coiled coils; green bars, highly conserved stretches in the C-termini. **b** The N-terminus of Xyr1 fused to the C-termini of Ypr1 and Ypr2 resulting in the fusion TFs XY1 and XZ0b, respectively

Having the correct sequences of Ypr1 and Ypr2 at hand, we performed a conserved domain search using the NCBI conserved domain database [45]. Next, we identified the highly conserved regions within the C-terminal parts of each of the three TFs by performing a BLAST analysis on the NCBI server [46] and a consecutive multiple sequence alignment (COBALT) [47] with their respective homologs. Further, we searched for coiled coils using the ExPASy Portal [48]. The obtained results are depicted in Fig. 9a. All three TFs contained a Gal4-like Zn(II)2Cys6 binuclear cluster DNA-binding domain (smart00066) at the N-terminus, a FTFMHR (cd12148) spreading approximately over the C-terminal half of the proteins. Within the FTFMHR, two further conserved domains were predicted, i.e., the two fungal-specific transcription factor domains, pfam04082 and smart00906 (Fig. 9a). All three TFs contain at least one predicted coiled-coil region, but none of them are at the same relative location (Fig. 9a). However, in all three TFs, the region directly in front of the FTFMHR is highly conserved, as are most parts of C-termini. We, therefore, decided to use the C-terminal parts starting at the conserved region in front of the FTFMHR for the protein domain exchanges.

### Integration of the fusion transcription factors XY1 and XZ0b into the Xyr1-deficient strain

We constructed the two fusion TFs, XY1(Xyr1::Ypr1) and XZ0b (Xyr1::Ypr2) by fusing the N-terminal part of Xyr1 (aa 1–336) to the C-terminal part of Ypr1 (aa 185–675) and Ypr2 (aa 204–684), respectively (Fig. 9b). The expression cassettes for the fusion TFs XY1 and XZ0b were inserted into the *pyr4* locus analogously to *xyr1* (Fig. 3a) by transforming pRP4-TXY(1) and pRP4-TXZ(0b) in the Xyr1-deficient strain Xyr1′(81), resulting in the strains TXY(1) and TXZ(0b), respectively. We confirmed the correct and exclusive integration of the expression cassettes at the *pyr4* locus by PCR and Southern blot analysis (Fig. 3b, c). Overexpression of the fusion TFs was verified by a RT-qPCR assay using *xyr1* primers (targeting the functional DNA-binding domain, which is part of the fusion TFs, but not the truncated Xyr1) and as template cDNA derived from mycelium samples grown on MEX plates. The levels were in both strains approx. tenfold higher than in the wild-type-like strain *T. reesei* Δ*tmus53.*

### The fusion transcription factors XY1 and XZ0b complement Xyr1 deficiency

**Fig. 10:**
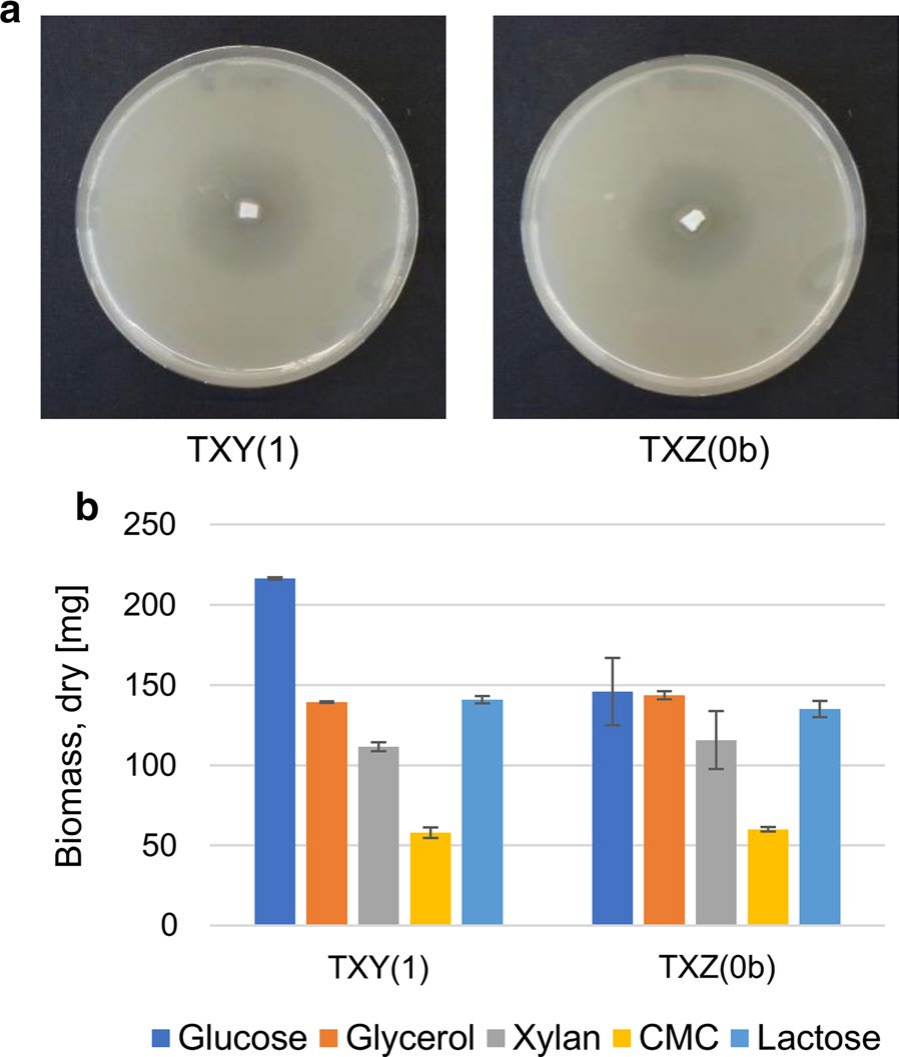
Phenotype of strains bearing TFs XY1 and XZ0b. **a** The *T. reesei* strains TXY(1) and TXZ(0b) were cultivated on xylan plates and pictures were taken after 72 h. **b** The *T. reesei* strains TXY(1) and TXZ(0b) were cultivated on the indicated carbon sources for 72 h and the dry weight of the accumulated biomass was measured. The values provided in the figures are means from three biological experiments. Error bars indicate standard deviations

To test the regulatory properties of the fusion TFs, XY1 and XZ0b, the two strains bearing the respective expression cassettes, TXY(1) and TXZ(0b) were grown on xylan plates. Both strains were able to form halos which indicates that the TFs are transactivating the expression of xylanases (Fig. 10a). Next, we cultivated the two strains on the carbon sources that were previously used to assess the Xyr1-overexpression strain TX(WT), i.e., glucose, glycerol, xylan, CMC, and lactose. After 72 h of cultivation, we measured the accumulated biomass and the enzyme activities in the resulting supernatants. Both strains were able to grow on xylan, CMC, and lactose (Fig. 10b) and exhibited xylanolytic and cellulolytic activity on all carbon sources tested (Figs. 5, 6). As a tendency, TXY(1) produced higher amounts of enzymes compared to TXZ(0b).

TXY(1) produced approx. 2500 U/g endo-xylanase activity on xylan, which is in the same range as on glucose and CMC (approx. 2000 U/g; Fig. 5a). In comparison, TX(WT) produced approx. 4500 U/g on xylan but none on glucose and on glycerol (Fig. 5a). We observed outstanding endo-xylanase production rates of TXY(1) on glycerol, namely 10,000 U/g, which is over two times higher than the highest expression rate of TX(WT) (approx. 4500 U/g on xylan; Fig. 5a). On lactose, TXY(1) secreted approx. 4500 U/g endo-xylanases (Fig. 5a). Notably, the wild-type-like Δ*tmus53* and the Xyr1-overexpression strain TX(WT) do not produce xylanases in amounts worth mentioning on any carbon source other than xylan (Fig. 5a). The strain TXZ(0b) produced endo-xylanases nearly carbon source independent with approx. 1500 U/g on glycerol, xylan, CMC, and lactose—only on glucose lower amounts were measured (approx. 600 U/g) (Fig. 5a). The general expression pattern is very similar for the β-xylosidase BXLI (Fig. 5b). Again, outstandingly high enzyme activity was observed in TXY(1), on all tested carbon sources (Fig. 5b). The highest levels were measured on glycerol (approx. 650 U/g), the lowest on xylan and CMC (approx. 100 U/g). For comparison, the highest ß-xylosidase activity of the Xyr1-overexpression strain TX(WT) was approx. 70 U/g on xylan (Fig. 5b).

TXZ(0b) produced endo-xylanase activity nearly carbon source independently (approx. 1500 U/g on glycerol, xylan, CMC, and lactose compared to approx. 600 U/g on glucose; Fig. 5a). β-xylosidase activity was detected on glycerol, xylan, and lactose in amounts worth mentioning (Fig. 5b).

Analogously, TXZ(0b) produced similar amounts of cellulases on nearly all carbon sources (approx. 5 arbitrary U/g total cellulase activity and 30 U/g endo-cellulase activity on all carbon sources other than glycerol, where we detected approx. 10 U/g endo-cellulase activity (Fig. 6a, b). The strain TXY(1) produced high amounts of total and endo-cellulase activity on lactose, where the Xyr1-overexpression strain TX(WT) failed to produce any (Fig. 6a, b) We detected also high total cellulases activity on glycerol, but only low levels on CMC and xylan (Fig. 6a). On glucose, the total cellulase activity was similar in TXY(1) compared to TX(WT) (Fig. 6a). The carbon source-dependent production pattern is similar for the endo-cellulolytic activity: high levels on lactose and glycerol, and lower on xylan and CMC (Fig. 6b).

The β-glucosidase activity was induced by the fusion TF XY1 on glucose, glycerol, and CMC, and to minor extent also on lactose, when compared to the basal enzyme activity levels in Δ*tmus53*, Xyr1′(81), and TX(WT) (Fig. 6c). In TXZ(0b), a higher basal enzyme activity levels could be observed (Fig. 6c).

### Regulatory influence of the fusion transcription factor XY1 on transcript levels of PCWDE-encoding genes

The strain TXY(1) exhibited strong xylanolytic and cellulolytic activity on the two cheap carbon sources glycerol and lactose. Therefore, we were interested in the regulatory influences of the fusion TF XY1 on the expression of individual PCWDEs. Consequently, we cultivated the strain TXY(1) on glucose, glycerol, xylan, CMC, and lactose and took samples at early stages of cultivation (48 h for CMC and 24 h for the other) to determine the transcript levels of the main PCWDEs. We isolated the total RNA from the samples and reverse transcribed the mRNA to perform qPCR assays. We determined the relative transcript levels for the PCWDE-encoding genes *cbh1*, *cbh2*, *egl1*, *bgl1*, *xyn1*, *xyn2*, *bxl1*, and the aldose reductase *xyl1* (Figs. 7, 8). Again, all samples were normalized to the glycerol sample of the wild-type-like Δ*tmus53*. We observed generally high transcript levels of the tested genes in a nearly carbon source independent manner (Figs. 7, 8). The transcript levels of *cbh1*, *cbh2*, *xyn2*, *bxl1*, and *xyl1* in TXY(1) were in the same range as the respective highest levels detected in TX(WT) (Figs. 7, 8). The transcript levels of *xyn1* were substantially higher (approx. 2 orders of magnitude) in TXY(1) on all carbon sources compared to the induced levels in Δ*tmus53* and TX(WT) (Fig. 8). On the other hand, XY1 could not induce the transcription of *egl1* that strongly (Fig. 7). Transcription of *egl1* was only induced on glucose and lactose (Fig. 7). The transcript levels of *bgl1* where generally higher in TXY(1) than in the other tested strains (Fig. 7).

## Discussion

In previous studies, Xyr1 as the main activator of cellulases and xylanases was overexpressed with the aim to enhance enzyme production yields [31, 32]. Following the same objective, the transactivation domain of VP16 was fused to Xyr1 in another study [34]. These experiments were performed in Xyr1-positive backgrounds, because until now, no *xyr1* deletion strain could be fully reconstituted. In this study, we aimed to construct a Xyr1-deficient platform strain for Xyr1-related studies and overexpression of fusion TFs without any possible heterodimer formation. Further, we followed overexpression strategies of wild-type Xyr1 and fusion TFs containing the DNA-binding domain of Xyr1 and the transactivation domain of Gal4-like TFs from a secondary metabolite biosynthetic gene cluster.

In some cases, the measured transcript levels did not match the finally reached enzymatic activities (e.g., transcript levels of cellulase-encoding genes and cellulase activity in TX(WT) on glycerol (Figs. 6b, 7). We want to stress that the transcript levels were measured only at a single time point. They do not represent the transcript levels throughout the complete cultivation period. A potential decrease of the transcript level at later time points was not detected. However, the transcript levels were meant to quantify the inducibility at an early time point to reflect the influence of the used TFs on inducibility.

We did not normalize the β-glucosidase activity to the acquired biomass in Fig. 6c, because this would result in seemingly high enzyme activities in the Xyr1-deficient strain on xylan and lactose, where this strain has a growth deficiency (Fig. 2a). We consider the measured β-glucosidase activities to be caused by the expression of other obviously Xyr1-independent *bgl* genes (e.g., *bgl2*) leading to a constitutive basal enzyme activity formation [49].

The overexpression of Xyr1 enhanced the production rates of xylanases on xylan and those of cellulases on xylan and CMC. In contrast, the cultivation of the Xyr1-overexpression strain TX(WT) on lactose resulted in a severely reduced cellulase production. This result was very surprising as lactose is considered to be an inducer of cellulases expression. However, it was previously suggested that the induction mechanism of cellulase expression is different on lactose than on cellulosic material. In a recent study, the mating-type locus protein Mat1-2-1 was shown to be necessary for cellulase production on lactose [21]. Zheng et al. further suggested that Mat1-2-1 was interacting with Xyr1. Based on this assumption, we speculate that an imbalance between Mat1-2-1 and Xyr1 in TX(WT) might lead either to a titration or a site occupation effect preventing Mat1-2-1 from participating in the induction of cellulases expression. Notably, both, TXY(1) and TXZ(0b) were able to produce cellulases on lactose, demonstrating that cellulase expression can be induced on lactose in the Xyr1′(81) background and that the inability of TX(WT) to produce cellulases on lactose is a specific attribute of the FTFMHR of Xyr1.

In the strain TXY(1), outstandingly high xylanase expression levels could be obtained on glycerol. Notably, glycerol is a cheap carbon source because it is a major co-product of biodiesel production and thereby influences the chain sustainability of the production process [50]. The application of the strain TXY(1) or of the fusion TF XY1 in any high-yielding *T. reesei* strain might offer a novel opportunity for glycerol utilization. Similarly, XY1 strongly induced cellulase production on glycerol and lactose. We speculate that the application of XY1 in a high-yielding strain would further push the production rates. Additionally, XY1 upregulated the expression of β-glucosidase on all tested carbon sources, with the highest measured enzyme activities on glycerol. The low amount of β-glucosidase secreted by *T. reesei* is considered to be the limiting factor during cellulose saccharification [51]. The utilization of the fusion TF XY1 might help to overcome this problem. However, the fusion TF XY1 has its limitations and its potential utilization must be put into the context of the particular aim. For instance, the over-expression of wild-type Xyr1 leads to higher cellulolytic activity on xylan and CMC and higher xylanolytic activities on xylan compared to the overexpression of XY1.

In the strain TXZ(0b), xylanase and cellulase production was successfully induced, indicating that the FTFMHR of Ypr2 possesses indeed transactivating properties. This was an open question resulting from a study on the regulatory system of the sorbicillinoid biosynthetic gene cluster [36]. There, a deletion of *ypr2* resulted in enhanced sorbicillinoid production rates. Suggesting that Ypr2 might be a repressor despite its Gal4-like nature. Here, we could demonstrate that Ypr2 contains a transactivating domain. Consequently, we suggest that Ypr2 activates the expression of a repressor, which then downregulates sorbicillinoid biosynthesis.

In both cases, the transactivation domain replacement in Xyr1 resulted in functional TFs, that changed the carbon source dependency of cellulase and xylanase production in *T. reesei*. Notably, these experiments were performed in a QM6a background, with Cre1 being intact. Naturally, the overexpression of the TFs suspends the influence of Cre1 on the level of *xyr1* expression, but CCR can take place on the level of cellulase and xylanase gene expression. The fusion TFs could successfully overcome CCR on that level as well, whereas the overexpression of Xyr1 resulted in only low cellulolytic and xylanolytic activities on glucose and glycerol. This suggests the presence of a carbon source-dependent auto-regulatory mechanism of Xyr1 located within the FTFMHR. Future studies might address this issue as well as further optimizations of the fusion TFs (*e.g*., influence of the linker region between DNA-binding domain and FTFMHR, addition of another autoregulatory domain that allows inducibility regardless of the carbon source used).

## Conclusions

Xyr1 overexpression is not an overall successful strategy to enhance cellulase and xylanase production, because it improves enzymatic production rates only on respective inducing carbon sources but cannot induce enzyme production on non-inducing carbon sources.

The fusion TF XY1 could successfully induce transcription of the main PCWDEs encoding genes nearly carbon source independently, allowing xylanase production while simultaneously utilizing the biodiesel co-product glycerol.

The fusion of the DNA-binding domain of one Gal4-like TF to the FTFMHR of another Gal4-like TF is a convenient strategy to draw conclusions on the regulatory properties of the involved TFs. In this case, the transactivating properties of Ypr2 could be verified.

## Methods

### Fungal strains and cultivation conditions

All *T. reesei* strains (Table 1) used in this study were maintained on malt extract agar at 30 °C. Uridine and Hygromycin B were added when applicable to a final concentration of 5 mM and 113 U/ml, respectively.

**Table 1:** *Trichoderma reesei* strains used in this study

Designation	Description	Source
Δ*tmus53*	Wild-type-like strain with deficiency of the non-homologous end joining repair pathway	[57]
Δ*pyr4*	Deletion of *pyr4* and its promoter in Δ*tmus53*	[44]
Xyr1′(81)	Insertion of a non-sense mutation at position 81 in Xyr1 in Δ*pyr4*	This study
TX(WT)	Xyr1-overexpressing strain; the wild-type *xyr1* under the control of the *tef1* promoter inserted at the *pyr4* locus, uridine prototrophy re-established	This study
TXY(1)	Overexpression of the fusion TF XY1; the fusion gene under the control of the *tef1* promoter is inserted at the *pyr4* locus, uridine prototrophy re-established	This study
TXZ(0b)	Overexpression of the fusion TF XZ0b; the fusion gene under the control of the *tef1* promoter is inserted at the *pyr4* locus, uridine prototrophy re-established	This study

For cultivations in shake flasks, *T. reesei* was grown in 50-ml Mandels–Andreotti (MA) medium [52] containing 1% (w/v) glucose monohydrate, glycerol, xylan from beechwood (Carl Roth GmbH + Co KG, Karlsruhe, Germany), CMC, or lactose at 30 °C on a rotary shaker at 180 rpm. Mycelia and supernatants were separated by filtration through Miracloth (EMD Millipore, part of Merck KGaA, Darmstadt, Germany). Mycelia were dried at 80 °C over night for biomass determination and supernatants were stored at − 20 °C.

For cultivations on xylan plates, *T. reesei* was pre-grown on MA medium plates containing 1% (w/v) xylan from beechwood (Roth) at 30 °C for 3 days in darkness. Then, an overgrown piece of agar was transferred to a fresh plate containing additionally 0.1% (v/v) Igepal and the plates were incubated at 30 °C in darkness.

### Plasmid constructions

PCRs for cloning purposes were performed with Q5 High-Fidelity DNA Polymerase (New England Biolabs, Ipswich, MA, USA) according to the manufacturer’s instructions. All used primers are listed in Table 2. PCR products were cloned into *Eco*RV-digested pJET1.2 (Thermo Scientific, part of Thermo Fisher Scientific Inc., Waltham, MA, USA) and after verification of the PCR products by sequencing (Microsynth, Balgach, Switzerland), they were released for subsequent cloning purposes by digestion with suitable restriction endonucleases (NEB). Synthesis of cDNA as templates for PCRs was carried out using the RevertAid™ H Minus First Strand cDNA Synthesis Kit (Thermo Scientific) according to the manufacturer’s instructions.

**Table 2:** Primers used in this study

Name	Sequence (5′–3′)
5Xyr1_fwd	TGTACATGTATGATGGCGTGC
Xyr1*_250rev-BamHI	CAGTACCCGTTGAATGGATCCTCTACCTGGCAGCAATAAGAGAGC
Xyr1*_250fwd-BamHI	CTTATTGCTGCCAGGTAGAGGATCCATTCAACGGGTACTGCTGGG
TXyr1_rev-KpnI	GGTACCATCAAGCCCTCTTCACTTTCAGC
Xyr1_3fwd-KpnI	GTACCGGGCTTGATTCACAGAATGATTC
Xyr1_3rev-NotI	GCGGCCGCTTCTTCTACTTCAAAGCTTTGGCAG
Ppki_5fwd	AGATAACGGTGAGACTAGCGGC
Tcbh2_rev_BcuI	ACTAGTGCTATTAACGTTTGGAAAGCCATC
Ptef_fwd-BspEI	TCCGGAGAGTTGGGCAAAATCAGGC
Ptef_rev-MCS	ACTAGTCTACGCTAGCGACCCATATGGATCTTAAGTGTGATGTAGCGTGAGAGCTG
Xyr1-fwd-NdeI	CATATGTTGTCCAATCCTCTCCGTC
Xyr1-rev-NheI	GCTAGCTAGAGGGCCAGACCGGTTC
Xyr1_P336r-MCS	GCTAGCGACCTCCAATTGCTCCGACGTCGCCATTAATGGGCTGCGAGAGCTG
Ypr1_L185f-MunI	CAATTGTTCTTACTCCACAGTCGACAACG
102499_rev-NheI	GCTAGCCGTAAATGCTCCCATC
Ypr2_P204f-MunI	CAATTGTTCCCCGGAATGTTGTGCTC
Ypr2_rev-SpeI	ACTAGTCTAGTAGTTCGTTCTCCTTCCAGTG
5Xyr1_fwd2	CGGAAGATACGATGGAGGAAC
Xyr1wt_Test_250rev	GCAGTACCCGTTGAATTCTTC
Xyr1*_Test_250rev	TACCCGTTGAATGGATCCTCTAC
5pyr4_fwd3	CCAGACGGTGATTCACATATACG
Ptef_rev-BspTI	CTTAAGTGTGATGTAGCGTGAGAGCTG
pyr4_3fwd	AGACGAGGACCAGCAGACC
Tpyr4_rev2	CAGGAAGCTCAGCGTCGAG
Xyr1_1760rev-NotI	GCGGCCGCGTTCAAGTCGTGCTCATCCAC
5pyr4_fwd(BglII)	GCGGAAGATCTCGAGATAGTATCTC
5pyr4_rev-BspEI	TCCGGAGTAGCTCTTCACTGGTTGTGGTG
sar1fw	TGGATCGTCAACTGGTTCTACGA
sar1rev	GCATGTGTAGCAACGTGGTCTTT
act1f	TGAGAGCGGTGGTATCCACG
act1r	GGTACCACCAGACATGACAATGTTG
xyr1_q2f	TCCGTCGCTATTCTGCCTAC
xyr1_q2r_wt_1	CAGCAGTACCCGTTGAATTC
cbh1f	GATGATGACTACGCCAACATGCTG
cbh1r	ACGGCACCGGGTGTGG
cbh2f	CTATGCCGGACAGTTTGTGGTG
cbh2r	GTCAGGCTCAATAACCAGGAGG
egl1f	CTGCAACGAGATGGATATCCTGG
egl1r	GTAGTAGCTTTTGTAGCCGCTGC
bgl1_q1f	ATCATTCTGGAGCAGATTCTTG
bgl1_q1r	GTAAGACAGTCCATAGCCGAAC
xyn1f	CAGCTATTCGCCTTCCAACAC
xyn1r	CAAAGTTGATGGGAGCAGAAG
xyn2_q1f	CCGTCAACTGGTCCAACTCG
xyn2_q1r	GTGCGGTAAATGTCGTAGACG
bxl1_q1f	GAATGACATGAACCTCCGACC
bxl1_g1r	CGAAGGTGAAGACGGGAATC
xyl1-fwd	CTGTGACTATGGCAACGAAAAGGAG
xyl1-rev	CACAGCTTGGACACGATGAAGAG

To introduce the non-sense mutation at position 81 into Xyr1, we constructed the plasmid pCD-Xyr1′(81)-HR using the following strategy: first, the promoter and the 5′part of *xyr1* were amplified by PCR using the primers 5Xyr1_fwd and Xyr1*_250rev-BamHI and chromosomal DNA of *T. reesei* Δ*tmus53* as template. This constitutes the 1.65 kb long 5′flank for the subsequent homologous recombination (Fig. 1a, left green box). The PCR product was inserted into pJET1.2 in the same direction as *eco47IR*, resulting in the plasmid pJET-5′xyr1. In parallel, the remaining part of *xyr1* and the terminator sequence were amplified by PCR using the Primers Xyr1*_250fwd-BamHI and TXyr1_rev-KpnI and chromosomal DNA of *T. reesei* Δ*tmus53* as template, and then inserted into pJET1.2 in the same direction as *eco47IR*. Next, the 765-bp-long 3′flank of *xyr1* (Fig. 1a, right green box). was amplified by PCR using the primers Xyr1_3fwd-KpnI and Xyr1_3rev-NotI and chromosomal DNA of *T. reesei* Δ*tmus53* as template, and the inserted into the previous plasmid via digestion with *Kpn*I and *Not*I. The *Bam*HI/*Not*I fragment from the resulting plasmid was inserted into *Bam*HI/*Not*I-digested pJET-5′xyr1, resulting in the plasmid pJET-xyr1Loc*. Finally, a hygromycin resistance cassette was amplified using the primers Ppki_5fwd and Tcbh2_rev_BcuI and pRLM_ex_30 [53] as template. The PCR product was directly inserted into pJET-xyr1Loc* that was previously digested with *Kpn*I and treated with blunting enzyme from the CloneJET PCR Cloning Kit (Thermo Scientific). The hygromycin resistance cassette is also functional in *E. coli* and the insertion could, therefore, can be selected for. The orientation of the hygromycin cassette was determined by sequencing (Fig. 1a, yellow arrow).

To overexpress Xyr1, we constructed the plasmidpRP4-TX(WT) using the following strategy: first, the promoter of *tef1* was amplified with the primers Ptef_fwd-BspEI and Ptef_rev-MCS and chromosomal DNA of *T. reesei* Δ*tmus53* as template and inserted into *Eco*RV-digested pJET1.2 resulting in pJET-Ptef(MCS). Next, the coding sequence of *xyr1* was amplified using the primers Xyr1-fwd-NdeI and Xyr1-rev-NheI and as template cDNA of *T. reesei* Δ*tmus53* grown on lactose. The *xyr1* coding sequence was inserted into pJET-Ptef via digestion with *Nhe*I and *Nde*I. The Ptef::*xyr1* fragment was released from the resulting plasmid by digestion with *Kpn*2I and *Spe*I and inserted into accordingly digested pCD-RPyr4T [44].

To overexpress the fusion TF XY1, we constructed the plasmid pRP4-TXY(1) using the following strategy: first, the coding sequence for the N-terminal part of Xyr1 was amplified by PCR using the primer Xyr1-fwd-NdeI and Xyr1_P336r-MCS and as template cDNA of *T. reesei* Δ*tmus53* grown on lactose. The gene part was inserted into pJET-Ptef(MCS) via digestion with *Nde*I and *Nhe*I resulting in the plasmid pJET-Ptef-xyr1 N. Next, the coding sequence for the C-terminal part of Ypr1 was amplified by PCR using the primer Ypr1_L185f-MfeI and 102499_rev-NheI and as template cDNA of *T. reesei* Δ*tmus53* grown on glucose, and then inserted into pJET-Ptef-xyr1 N via digestion with *Mfe*I and *Nhe*I. The Ptef::*xyr1*::*ypr1* fragment was released from the resulting plasmid by digestion with *Kpn*2I and *Spe*I and inserted into accordingly digested pCD-RPyr4T [44].

To overexpress the fusion TF XZ0b, we constructed the plasmid pRP4-TXZ(0b) using the following strategy: first, the coding sequence for the C-terminal part of Ypr2 was amplified by PCR using the primer Ypr2_P204f-MunI and Ypr2_rev-SpeI and as template cDNA of *T. reesei* Δ*tmus53* grown on glucose, and then inserted into pJET-Ptef-xyr1 N via digestion with *Mfe*I and *Spe*I. The Ptef::*xyr1*::*ypr2* fragment was released from the resulting plasmid by digestion with *Kpn*2I and *Spe*I and inserted into accordingly digested pCD-RPyr4T [44].

### Fungal transformations

The protoplast generation and transformation of *T. reesei* was performed as described previously [54]. Typically, 10 µg of linearized plasmid DNA (in 15-µl sterile ddH_2_O) was used for the transformation of 10^7^ protoplasts (in 200 µl). Selection was performed as described previously [44]. Resulting candidates were subjected to homokaryon purification by streaking conidia on selection plates.

### Isolation of chromosomal DNA

Chromosomal DNA was isolated from mycelium by grinding in liquid nitrogen followed by a phenol/chloroform extraction [54]. RNA was degraded using RNaseA (Thermo Scientific). DNA was precipitated with isopropanol, washed with 70% ethanol, and dissolved in ddH_2_O.

### Genotype testing by PCR

For testing the genotype, 10 ng of chromosomal DNA was used as template in a 25-µl PCR using OneTaq polymerase (NEB) according to the manufacturer’s instructions. All used primers are listed in Table 2.

### Southern blot analysis

15 µg of chromosomal DNA was digested with 30 U of the given restriction enzymes (NEB). The resulting DNA fragments were separated by electrophoresis on an 0.8% agarose gel, then denatured in 0.4-M NaOH, and transferred by capillary forces onto a Biodyne B 0.45-µm nylon membrane (Pall Corporation, Port Washington, NY, USA) using 10 × SSC. 1.5 µg of biotinylated DNA probe was used for hybridization at 65 °C overnight. Probes were generated by PCR using the primers 5Xyr1_fwd and Xyr1_1760rev-NotI (Fig. 1a, c) or 5pyr4_fwd(BglII) and 5pyr4_rev-BspEI (Fig. 3a, c) using chromosomal DNA of *T. reesei* Δ*tmus53* as template. Labeling of the probe was performed using a Klenow Fragment (exo-) (Thermo Scientific), random hexamer primers, and biotin-11-dUTP (Jena Bioscience, Jena, Germany). Signals were visualized using Poly-HRP conjugated to streptavidin and ECL Plus Western Blotting substrate (both Thermo Scientific) on a ChemiDoc MP (Bio-Rad Laboratories, Hercules, USA).

### Determination of enzymatic activities

Total cellulolytic enzyme activity of cultivation supernatants was measured using the Cellulase Activity Assay kit (Fluorometric) (abcam189817, Abcam PLC, Cambridge, UK) according to the manufacturer’s instructions, with the following adoptions: fluorescence was measured on a Promega GloMax Multi Detection system using the green filter cube (Ex: 520 nm, Em: 580–640 nm), measured fluorescence change rate (Δfluo/min) was used to calculate arbitrary units/ml by multiplying Δfluo/min with 5.1136 * 10^−4^. Measurements were performed in technical duplicates.

Endo-xylanolytic and endo-celluloytic activities of cultivation supernatants were measured with Azo-Xylan and Azo-CMC (both Megazyme International Ireland, Wicklow, Ireland) according to the manufacturer’s instructions, respectively. One unit of activity is defined as the amount of enzyme required to release one μmol of reducing sugar equivalents per minute.

β-xylosidase and β-glucosidase activities of cultivation supernatants were measured with *p*-nitrophenyl β-d-xylopyranoside and *p*-nitrophenyl β-d-glucpyranoside (both Merck KGaA, Darmstadt, Germany) as described previously [55]. One unit of activity is defined as the amount of enzyme required to release 1 µmol of glucose reducing sugar equivalents per minute under the defined assay conditions.

### RNA extraction

0.01–0.03 g of harvested mycelia was homogenized in 1 ml of peqGOLD TriFast DNA/RNA/protein purification system reagent (VWR, part of Avantor Performance Materials, LLC, Radnor, PA, USA) using a FastPrep FP120 BIO101 ThermoSavant cell disrupter (Qbiogene, Carlsbad, US). RNA was isolated according to the manufacturer’s instructions, and the concentration was measured using the NanoDrop ONE (Thermo Scientific).

### Transcript analysis by RT-qPCR

1 µg of isolated RNA was subjected to a DNaseI treatment (Thermo Scientific) according to the manufacturer’s instructions and then reverse transcribed using the LunaScript RT SuperMix (NEB) also according to the manufacturer’s instructions. The cDNA was diluted 1:50 and 2 µl was used as template in a 15-µl reaction using the Luna Universal qPCR Master Mix (NEB) according to the manufacturer’s instructions. All reactions were performed in triplicates on a Rotor-Gene Q system (Qiagen, Hilden, Germany). Calculations of the relative transcript levels were performed according to the Double Delta Ct method [56] using the reference genes *sar1* and *act1* for normalization.

## Data Availability

All data and materials described are freely available for scientific and academic purposes upon request to the corresponding author.

## Abbreviations

CCR: carbon catabolite repression
CMC: carboxymethyl cellulose
FTFMHR: Fungal Transcription Factor Middle Homology Region
PCWDEs: plant cell wall-degrading enzymes
RT-qPCR: reverse transcription quantitative PCR
TF: transcription factor
qPCR: quantitative PCR
